# Pilot-Scale Downstream Processing of Recombinant Influenza Virus Vectors Expressing *Brucella* spp. Antigens Using an Integrated Membrane-Chromatography Purification Platform

**DOI:** 10.3390/vaccines14070626

**Published:** 2026-07-17

**Authors:** Nurika Assanzhanova, Aigerim Sagymbayeva, Gaukhar Shynybekova, Kamshat Shorayeva, Sholpan Ryskeldinova, Aigerim Mailybayeva, Yeldos Myrzakhmetov, Ekaterina Yamanova, Rassul Sidikhov, Meiyrim Almezhanova, Samat Zhaksylyk, Zharkynai Absatova, Kuandyk Zhugunissov, Olga Chervyakova, Nurlan Akmyrzayev

**Affiliations:** Research Institute for Biological Safety Problems, National Holding “QazBioPharm”, Gvardeiskiy 080409, Kazakhstan; n.assanzhanova@biosafety.kz (N.A.); a.sagymbayeva@biosafety.kz (A.S.); sh.gaukhar@biosafety.kz (G.S.); k.shorayeva@biosafety.kz (K.S.); sh.ryskeldinova@biosafety.kz (S.R.); a.mailybayeva@biosafety.kz (A.M.); y.myrzakhmetov@biosafety.kz (Y.M.); e.yamanova@biosafety.kz (E.Y.); sidikhovrb@mail.ru (R.S.); m.almezhanova@biosafety.kz (M.A.); s.zhaksylyk@biosafety.kz (S.Z.); zh.absatova@biosafety.kz (Z.A.); k.zhugunisov@biosafety.kz (K.Z.)

**Keywords:** brucellosis, recombinant vaccine, influenza virus, viral vector, virus purification, ion-exchange chromatography, tangential flow filtration, *Brucella*

## Abstract

**Background**: Brucellosis remains a significant zoonotic infection affecting approximately 500,000 people worldwide annually, with no licensed human vaccine available. Recombinant influenza virus vectors expressing *Brucella* spp. antigens represent a promising vaccine platform. However, transitioning from laboratory constructs to clinical candidates requires validation of scalable purification methods compliant with Good Manufacturing Practice (GMP) standards. This study aimed to develop and optimize a pilot-scale purification protocol for these vectors. **Methods**: Recombinant influenza A viruses (H5N1) expressing *Brucella* spp. antigens (Omp16, Omp19, L7/L12, Cu-Zn SOD) were propagated in MDCK cell culture. The optimized purification process included: (1) clarification; (2) ultrafiltration/diafiltration (100 kDa MWCO); (3) two-step chromatography (anion-exchange Q-Sepharose^®^ Fast Flow and multimodal Capto™ Core 700); and (4) sterile filtration. Process validation was performed across three independent pilot-scale batches (20 L each). **Results**: The purification process demonstrated high reproducibility for all constructs. Final preparations met established quality criteria: infectious titer ≥ 5.2 log_10_ TCID_50_/mL, hemagglutination activity 7.33 ± 0.58–8.33 ± 0.58 log_2_, total protein content 157–305 μg/mL, residual host cell DNA < 10 ng/dose, and bacterial endotoxin levels ≤ 0.15 IU/mL. The overall recovery of infectious virus was 20–24%, an optimal value for multi-stage bioprocessing. Preservation of the target genetic insert was confirmed in all final preparations by PCR and sequencing. **Conclusions**: The developed integrated purification protocol yields vectors with high purification efficiency, preserving biological activity and meeting regulatory quality requirements for residual host cell DNA and endotoxins. The technological platform demonstrated versatility, robustness (inter-batch coefficient of variation for yield did not exceed 10–12%), and scalability, establishing a foundation for preclinical and clinical studies of candidate brucellosis vaccines.

## 1. Introduction

Brucellosis is a widespread zoonotic disease transmitted to humans primarily through direct contact with infected animals or the consumption of contaminated animal products. The primary causative agents of human infection are *Brucella melitensis*, *B. abortus*, and *B. suis*, whose natural reservoirs are small ruminants, cattle, and pigs, respectively. Other species of the genus *Brucella* are of secondary importance in human pathology [1].

Infection typically occurs through the consumption of unpasteurized dairy products or contact with infected tissues (e.g., aborted placentas) without the use of personal protective equipment [2,3]. Occupational groups, including livestock workers, slaughterhouse employees, and veterinary professionals, are at an elevated risk [4]. Although brucellosis is rarely fatal, the disease often follows a chronic course, causing persistent disability and inflicting substantial socio-economic damage on endemic regions [5,6].

Globally, approximately 500,000 new human cases of brucellosis are reported annually [7], with the susceptible population estimated at 2.4–3.5 billion people [8,9]. Socioeconomic development and public health measures have significantly influenced the geographical distribution of the disease: incidence rates remain low in high-income countries, whereas the infection remains highly endemic in low- and middle-income countries [2]. In the Republic of Kazakhstan, according to epidemiological surveillance data from 2018 to 2023, brucellosis continues to predominantly affect the adult population, while the incidence among children and adolescents remains low and stable.

Despite significant advances in veterinary prophylaxis, there is currently no licensed vaccine for the prevention of human brucellosis. Live attenuated vaccines used in animal husbandry (*B. abortus* S19, *B. melitensis* Rev.1, *B. abortus* RB51) are contraindicated for human use due to residual virulence and the risk of disease development in immunocompromised individuals [10]. Consequently, modern vaccine development strategies are shifting toward subunit and viral vector platforms that combine an improved safety profile with the ability to induce robust cellular immunity, which is critically important for the elimination of intracellular pathogens [11,12].

In this context, recombinant influenza virus vectors are of particular interest. Key advantages of this platform include well-established manufacturing technologies, the possibility of intranasal administration (which ensures the induction of mucosal immunity at the portals of infection), and the capacity to stimulate a potent T-cell response [13,14]. Previous studies have demonstrated that the expression of immunodominant *Brucella* antigens (such as L7/L12, Omp25, or BP26) within a recombinant influenza virus confers protective immunity in experimental animal models [15].

However, the transition from laboratory constructs to clinical candidates requires the resolution of technological challenges associated with downstream processing. Traditional purification methods for viral vaccines, such as sucrose density gradient ultracentrifugation or polyethylene glycol precipitation, are characterized by several limitations: low yield of infectious virus, labor-intensiveness, scalability issues, and the risk of damaging viral particles, which may reduce the immunogenicity of the recombinant antigen [16,17]. To comply with Good Manufacturing Practice (GMP) requirements, purification methods must ensure a high degree of host cell DNA (HCD) removal and reduction of total protein impurities while preserving the biological activity of the virus [18]. A crucial step linking primary virus harvest and its final polishing is concentration and diafiltration. The application of tangential flow filtration (TFF) not only significantly reduces the volume of the process fluid but also optimizes the ionic strength and pH of the sample prior to chromatography, which is critical for the physicochemical stability of recombinant vectors.

In modern biotechnology, the purification of large and complex biological entities, such as recombinant viral vectors, critically depends on the selection of sorbents that provide high dynamic binding capacity and minimal impact on virion structure. Alongside the development of monolithic supports [19,20], next-generation high-throughput granular sorbents adapted for continuous flow operation and providing convective mass transfer are finding widespread application [21]. The use of a multi-stage purification strategy combining ion-exchange chromatography with multimodal polishing chromatography allows for high yields alongside the effective removal of host cell DNA and proteins. Nevertheless, the impact of such comprehensive processing conditions on the stability and functional expression of heterologous bacterial antigens (specifically, *Brucella* spp. antigens) within recombinant influenza virions remains unexplored, which hinders the standardization of the vaccine candidate manufacturing process.

Therefore, the aim of the present study was to develop and optimize a comprehensive downstream purification strategy for a recombinant influenza virus vector expressing *Brucella* spp. antigens, utilizing a sequential approach of tangential flow filtration, anion-exchange, and multimodal chromatography. The specific objectives were to: (1) determine the optimal conditions for primary concentration and diafiltration via TFF; (2) optimize the anion-exchange and multimodal flow chromatography steps to maximize vector yield and purity; (3) confirm the preservation of viral biological activity and the target genetic insert post-purification; and (4) evaluate the compliance of the final preparations with regulatory limits for residual host cell DNA and proteins.

## 2. Materials and Methods

### 2.1. Recombinant Viral Vectors

Recombinant influenza A viruses (subtype H5N1) expressing *Brucella* spp. antigens (Omp16, Omp19, ribosomal protein L7/L12, and Cu–Zn superoxide dismutase) were generated using an eight-plasmid reverse genetics system based on the pHW2000 vector [22]. The antigen-encoding genetic inserts were integrated in-frame at nucleotide position 80 of the non-structural protein 1 (NS1) gene.

Cell culture and virus propagation.

Virus propagation was performed using Madin-Darby Canine Kidney (MDCK) cells (ATCC, Manassas, VA, USA). Cells were maintained in OptiPRO™ SFM serum-free medium (Thermo Fisher Scientific, Waltham, MA, USA) at 34 °C in a 5% CO_2_ atmosphere. To ensure proteolytic activation of hemagglutinin, TPCK-treated trypsin (Sigma-Aldrich, St. Louis, MO, USA) was supplemented to a final concentration of 0.5 μg/mL. After 48 h of incubation, the culture supernatant was harvested and subjected to downstream processing.

### 2.2. Hemagglutination Assay

The hemagglutination (HA) assay was performed in 96-well U-bottom microtiter plates (Golias, Kranj, Slovenia; or Sterilin, Newport, UK), which represent the standard format for HA assays as they allow erythrocytes to settle into a distinct central ‘button’ in negative wells, facilitating clear visual discrimination of agglutination patterns. Serial two-fold dilutions of the samples in physiological saline were mixed with an equal volume of a 1% suspension of chicken erythrocytes.

### 2.3. TCID_50_ Assay

Viral infectivity across different fractions was determined by calculating the 50% tissue culture infectious dose (TCID_50_). Serial ten-fold dilutions of the samples were inoculated onto MDCK cells seeded in 96-well flat-bottom plates (Corning, New York, NY, USA). Cytopathic effect (CPE) was monitored after 72–96 h of incubation, and infectious titers were calculated using the Reed and Muench method [23] and expressed as TCID_50_/mL.

### 2.4. Purification of Virus-Containing Supernatant

**Figure 1:**
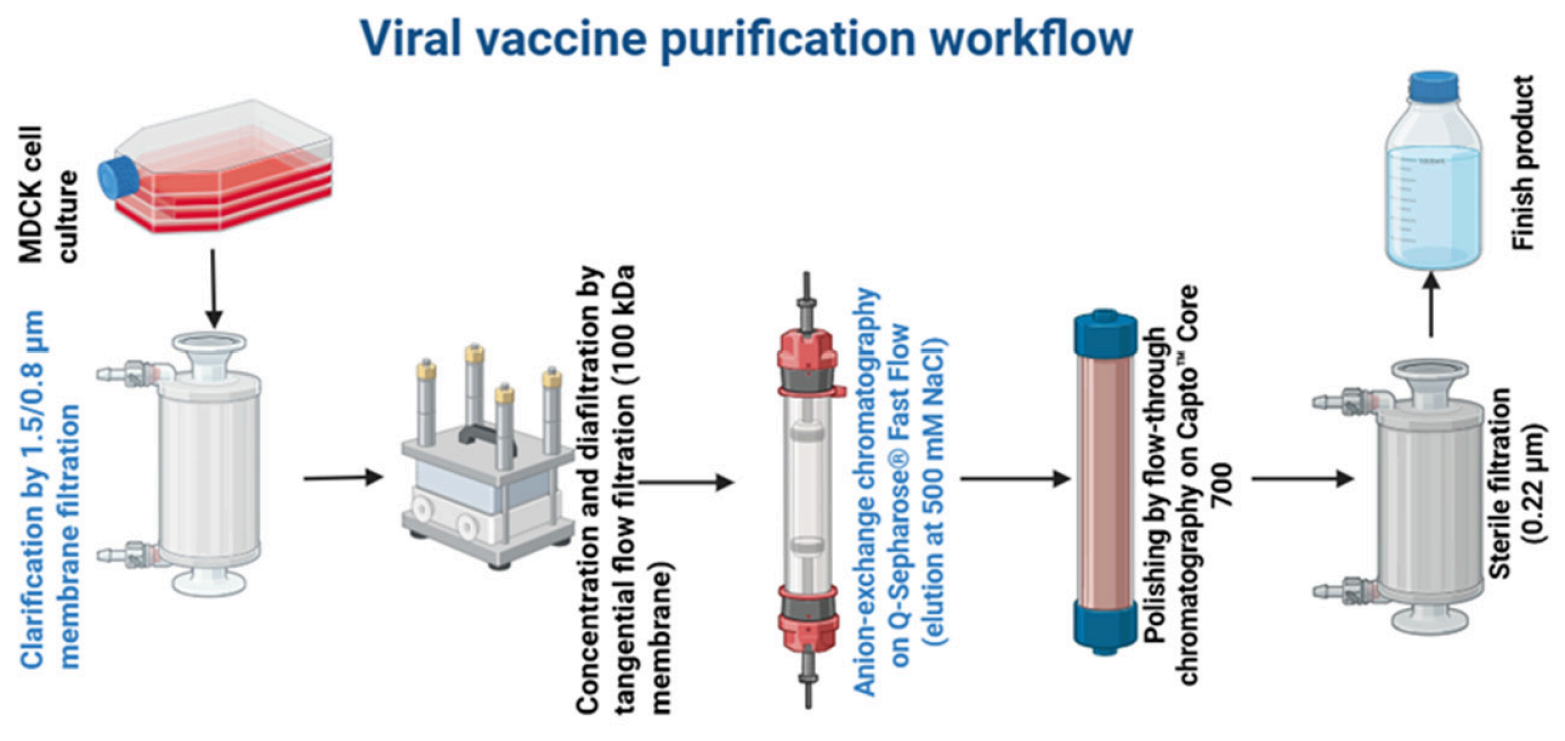
Schematic representation of the multi-stage downstream purification workflow for recombinant influenza viruses expressing *Brucella* spp. antigens. (Created with BioRender.com).

The process parameters for each purification step were selected based on preliminary laboratory-scale optimization and the physicochemical properties of influenza virions, with a focus on scalability and preservation of viral integrity. The criteria for optimization at the pilot scale included maximizing infectious virus recovery and minimizing residual impurities (total protein and rHCD) (Figure 1).

**Clarification and concentration.** The culture supernatant was clarified by sequential filtration through dual-layer capsule filters (1.5/0.8 μm, Cobetter, Hangzhou, China) at a pressure of 0.5–1.0 bar. Viral concentration was achieved via tangential flow filtration (TFF) using a Pellicon 2 cassette equipped with a Biomax^®^ 100 kDa membrane (Merck Millipore, Burlington, MA, USA) at a transmembrane pressure (TMP) ≤ 0.7 bar, reducing the volume 20-fold. Subsequent diafiltration was performed against 5–10 column volumes (CV) of 20 mM phosphate buffer (PB) containing 20 mM NaCl and 1 mM EDTA (pH 7.5). Process performance was monitored by HA activity, total protein concentration, and residual host cell DNA (rHCD) levels. Bacterial endotoxin testing and confirmation of viral biological activity were also performed following the concentration step.

**Chromatographic purification.** Primary capture was performed using anion-exchange chromatography on Q-Sepharose^®^ Fast Flow resin (Cytiva, Marlborough, MA, USA). After sample loading, the column was washed with equilibration buffer (20 mM PB, 100 mM NaCl, 1 mM EDTA, pH 7.5) to remove unbound impurities. The viral fraction was eluted using a stepwise salt gradient up to 500 mM NaCl in PBS supplemented with 1 mM EDTA (pH 7.5), continuing until the OD_280_ baseline stabilized. The linear flow velocity was maintained at 150 cm/h (corresponding to a volumetric flow rate of 20 mL/min for the column used).

Final polishing was performed on a Capto™ Core 700 multimodal resin (Cytiva) in flow-through mode. The purified target virus was collected in the column void volume, while impurities were retained within the matrix. At each purification stage, aliquots were collected for analytical testing. The working volumes were: initial culture supernatant (20 L); post-TFF concentrate (~1.0–1.2 L); Q-Sepharose^®^ chromatography (column bed volume of 900 mL); and Capto™ Core 700 polishing (column bed volume of 100 mL).

**Sterile filtration.** The purified viral preparation was sterile-filtered under aseptic conditions using a 0.22 μm vacuum filtration system (Filtermax, TPP Techno Plastic Products AG, Trasadingen, Switzerland). The final drug substance was characterized by a comprehensive quality control panel, including HA activity, infectious titer, total protein, residual host cell DNA, absence of bacterial endotoxins, and molecular confirmation of the target genetic insert.

Sterility testing during the purification stages was performed by direct inoculation into standard liquid media in accordance with pharmacopoeia guidelines. Samples were inoculated into fluid thioglycollate medium (FTM) (incubated at 30–35 °C for anaerobic and aerobic bacteria) and soybean-casein digest medium (tryptic soy broth, TSB) (incubated at 20–25 °C for fungi and aerobic bacteria) for 14 days to confirm the absence of microbial contamination.

### 2.5. Total Protein Quantification

Total protein concentration was quantified colorimetrically using the Lowry assay [24]. Briefly, proteins reacted with the Folin–Ciocalteu reagent (Sigma-Aldrich, Darmstadt, Germany) under alkaline conditions in the presence of copper ions to form a colored complex. Absorbance was measured at 750 nm using a UV-1900i UV-Vis spectrophotometer (Shimadzu Corporation, Kyoto, Japan), with bovine serum albumin (BSA; Thermo Fisher Scientific, Waltham, MA, USA) serving as the standard.

### 2.6. Quantification of Residual Host Cell DNA (rHCD)

Residual host cell DNA was quantified fluorometrically using the Quant-iT dsDNA Broad-Range Assay Kit (Thermo Fisher Scientific, Waltham, MA, USA), which utilizes a fluorescent dye that selectively binds to double-stranded DNA (dsDNA). Measurements were performed strictly according to the manufacturer’s protocol. The instrument was calibrated using two standard samples (0 and 10 ng/μL). Fluorescence intensity was recorded using a Qubit 4 Fluorometer (Thermo Fisher Scientific, Singapore) with excitation and emission wavelengths of approximately 500 nm and 525 nm, respectively. Final concentrations were calculated by accounting for the sample dilution factor.

### 2.7. Statistical Analysis

Statistical analysis was performed using GraphPad Prism software version 8.0 (GraphPad Software Inc., La Jolla, CA, USA). Data for infectious and hemagglutinating activity are expressed as mean ± standard deviation (SD). Statistical significance was defined as *p* < 0.05.

## 3. Results

### 3.1. Cultivation and Primary Characterization of Virus-Containing Supernatants

**Figure 2:**
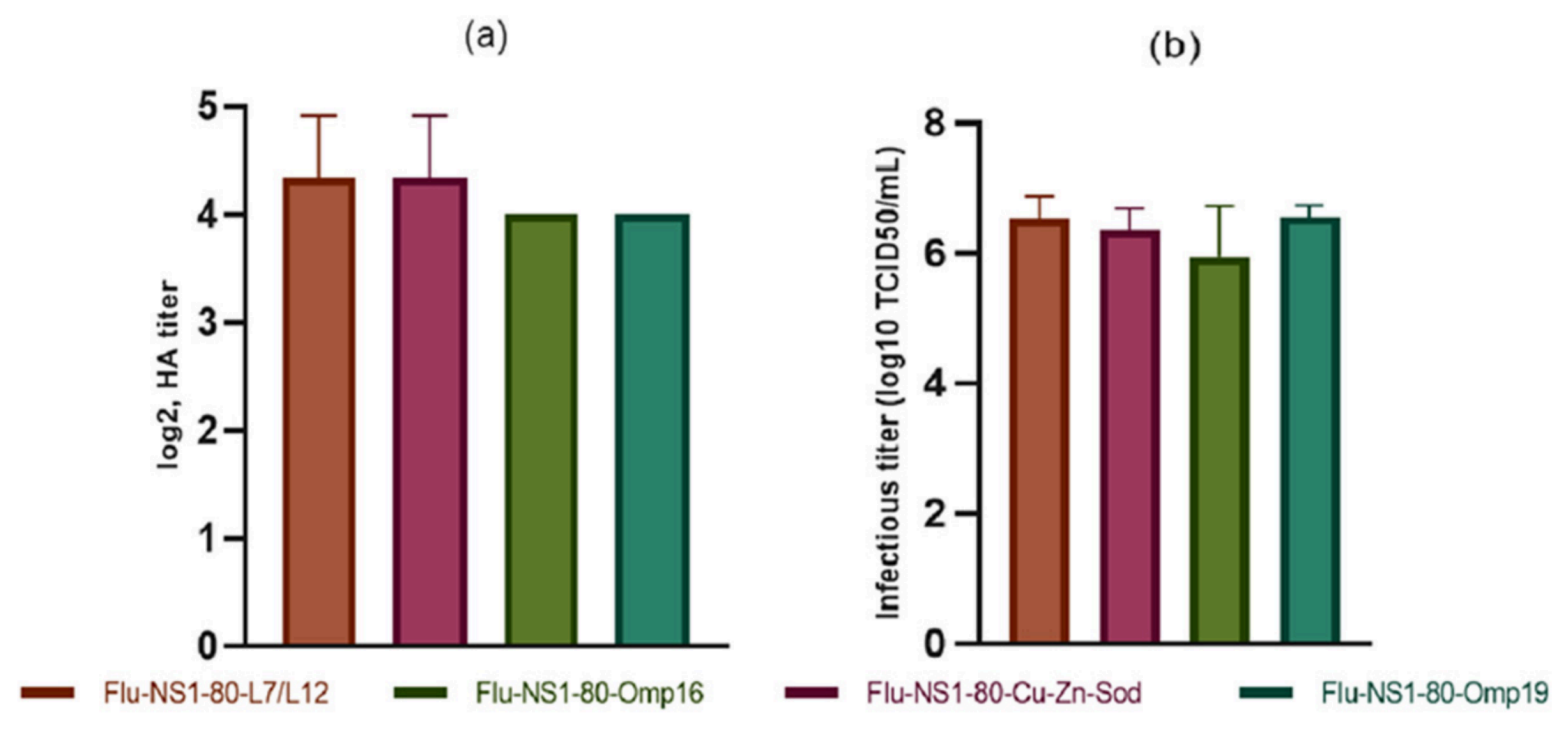
Comparative analysis of the initial viral activity of recombinant strains. (**a**) Hemagglutination activity; (**b**) Infectious titer. Data are presented as mean ± standard deviation (SD) of three biological replicates.

Pilot-scale cultivation was performed for each of the four recombinant strains (L7/L12, SOD, Omp16, and Omp19) to harvest the virus-containing supernatant (VCS). Experiments were conducted in three independent biological replicates (totaling 12 production runs) to evaluate process reproducibility. The initial parameters of the VCS regarding hemagglutination activity (HA) and infectious titer are presented in Figure 2.

As shown in Figure 2, HA remained stable across all samples, ranging from 4.00 ± 0.00 to 4.33 ± 0.58 log_2_, indicating uniform expression of surface antigens. The infectious titer varied depending on the strain, ranging from 5.94 ± 0.77 to 6.56 ± 0.17 log_10_ TCID_50_/mL, with inter-replicate variability not exceeding ±0.4 log_10_, confirming the reproducibility of the cultivation process.

### 3.2. Clarification by Membrane Filtration

Primary clarification of the VCS was performed using 1.5/0.8 μm capsule filters. HA titers remained at baseline levels, and fluctuations in the infectious titer were within the acceptable experimental error margin (≤0.2 log_10_). The stability of the infectious titers confirms the absence of viral inactivation, while the stable HA titers indicate the absence of non-specific adsorption or degradation of viral surface glycoproteins during membrane passage.

### 3.3. Concentration and Diafiltration by Tangential Flow Filtration (TFF)

**Figure 3:**
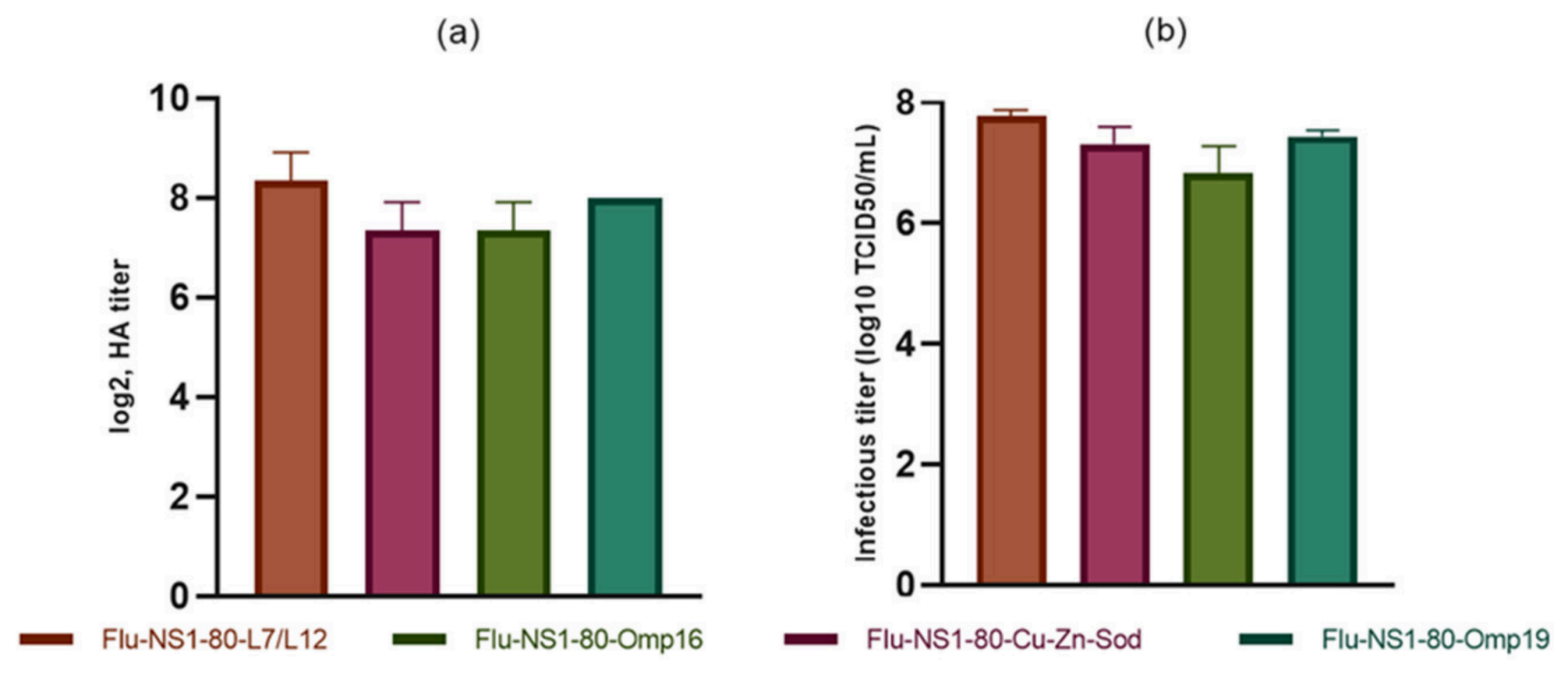
Dynamics of hemagglutination activity and infectious titer following tangential flow filtration. (**a**) Hemagglutination activity; (**b**) Infectious titer. Data are presented as mean ± SD of three biological replicates.

The clarified suspensions were subjected to concentration and diafiltration using Pellicon^®^ 2 cassettes (100 kDa MWCO). At this stage, a consistent increase in HA was recorded, reaching values of 7.33 ± 0.58 to 8.33 ± 0.58 log_2_, depending on the strain (Figure 3).

The increase in HA titers after TFF is attributed not only to the physical concentration of the virus but also to the effective removal of low-molecular-weight inhibitors of the hemagglutination reaction (salts, metabolites, protein fragments). The infectious titer post-diafiltration ranged from 6.83 ± 0.44 to 7.78 ± 0.09 log_10_ TCID_50_/mL, indicating the complete preservation of the vectors’ initial biological activity.

### 3.4. Two-Step Chromatographic Purification

#### 3.4.1. Anion-Exchange Chromatography on Q-Sepharose^®^ Fast Flow

**Figure 4:**
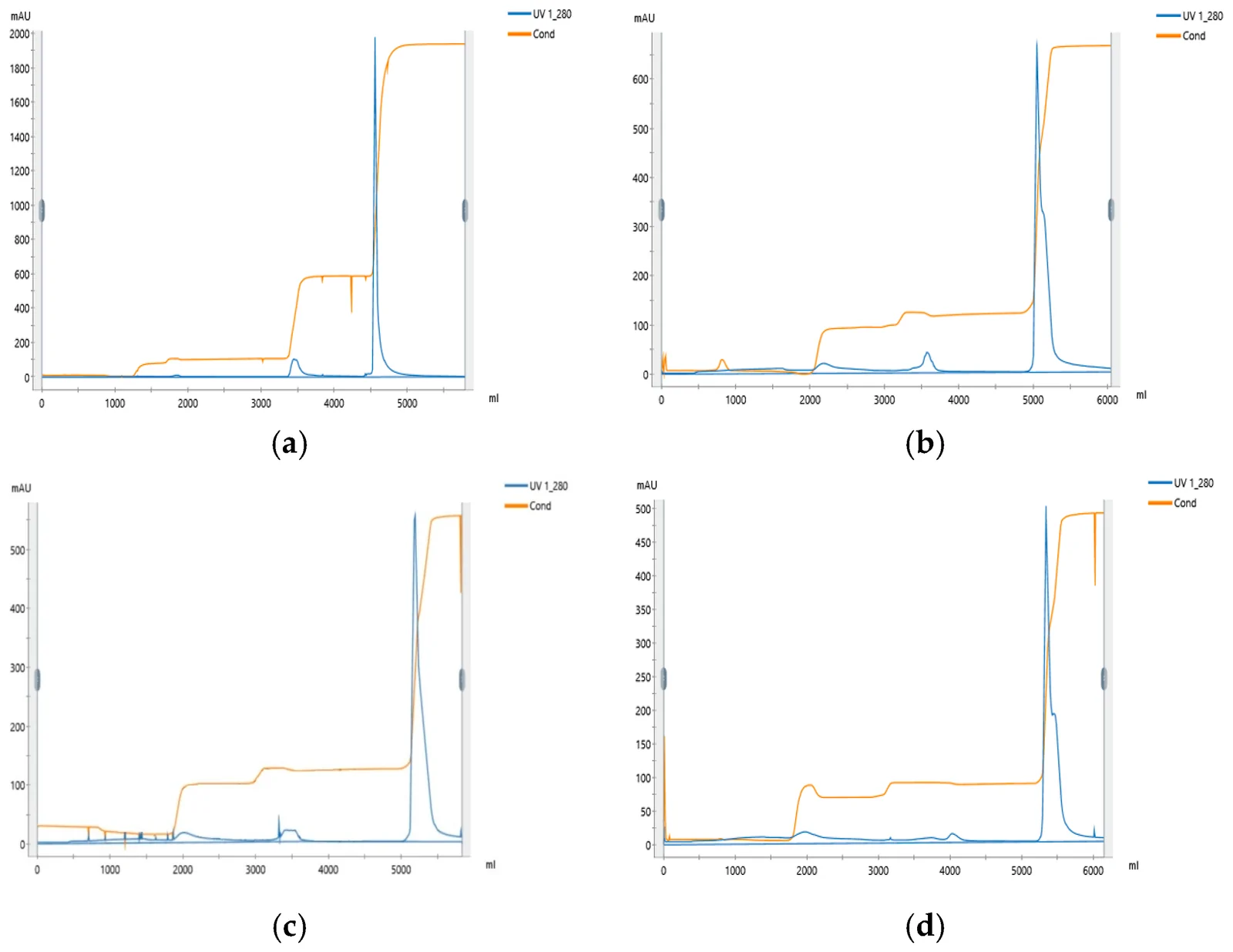
Chromatographic profiles of recombinant influenza virus purification on the Q-Sepharose^®^ Fast Flow anion-exchange resin. (**a**) Flu-NS1-80-SOD; (**b**) Flu-NS1-80-L7/L12; (**c**) Flu-NS1-80-OMP19; (**d**) Flu-NS1-80-OMP16. The *x*-axis represents eluate volume (mL), and the *y*-axis represents optical density at 280 nm (OD_280_, mAU). Arrows indicate chromatography steps: (1) sample loading; (2) washing with low-ionic-strength buffer (20 mM PB, 100 mM NaCl, 1 mM EDTA, pH 7.5); (3) elution with high-ionic-strength buffer (20 mM PB, 500 mM NaCl, 1 mM EDTA, pH 7.5). The main elution peak of viral particles is indicated by the shaded area. Note: The label ‘cond’ on chromatograms indicates the column conditioning/equilibration phase with starting buffer.

Primary purification of the viral concentrates was performed via anion-exchange chromatography (AEX) in a bind-elute mode. The chromatographic profiles of all four recombinant vectors demonstrated high reproducibility (Figure 4). During the loading and washing stages with a low-ionic-strength buffer (100 mM NaCl), the bulk of non-target host cell proteins and residual host cell DNA (rHCD) was effectively removed, while the viral particles were firmly retained on the resin.

The target virus was eluted as a narrow, symmetric peak upon increasing the salt concentration to 500 mM NaCl, which corresponds to the calculated isoelectric point of the virions. Minor differences in signal intensity (OD_280_) between strains are due to the specific nature of the expressed *Brucella* antigens and their effect on the overall particle charge. The selected mode allowed for a significant increase in the specific activity of the preparations with minimal loss of infectivity.

#### 3.4.2. Flow-Through Chromatography on Capto™ Core 700

**Figure 5:**
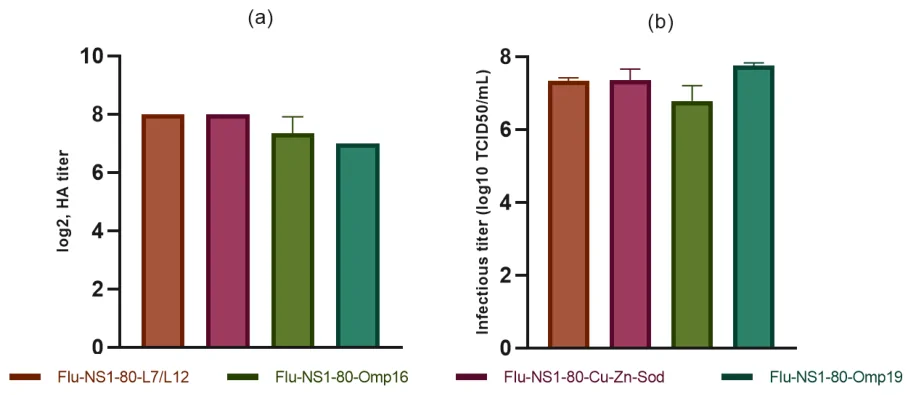
Characterization of recombinant influenza viruses following final polishing on Capto™ Core 700 resin. (**a**) Hemagglutination activity; (**b**) Infectious titer. Data are presented as mean ± SD of three biological replicates.

For final polishing, multimodal chromatography was performed using Capto™ Core 700 resin. Due to the size-exclusion effect, viral particles eluted in the column void volume, while residual proteins, DNA, and low-molecular-weight impurities were effectively retained within the internal matrix of the resin. This step significantly increased the specific activity of the concentrates, as evidenced by the maintenance of viral titers (Figure 5) alongside a substantial reduction in total protein concentration (Figure 7), while fully preserving HA and causing minimal loss of infectious titer (≤0.2 log_10_ TCID_50_/mL).

The dynamics of quality indicators upon completion of the final chromatographic purification are presented in Figure 5. The absence of statistically significant differences between strains in terms of hemagglutinating and infectious activity (*p* > 0.05, ANOVA) indicates the high reproducibility of the polishing process for all types of the investigated recombinant constructs.

### 3.5. Sterile Filtration

**Figure 6:**
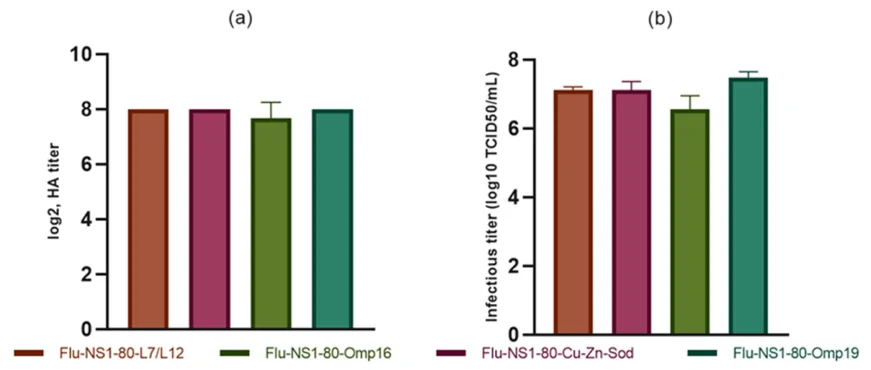
Characterization of recombinant influenza viruses following sterile filtration. (**a**) Hemagglutination activity; (**b**) Infectious titer. Data are presented as mean ± SD of three biological replicates.

The purified viral suspensions were subjected to sterile filtration through 0.22 μm membrane filters. This step was performed under aseptic conditions to ensure the microbiological purity of the vaccine candidates. It was established that the final filtration did not lead to statistically significant losses in biological activity: differences in HA and infectious titer (Figure 6) before and after filtration did not exceed 0.1–0.2 log_10_ (*p* > 0.05, ANOVA), confirming minimal adsorption of virions onto the membrane material.

### 3.6. Evaluation of Purification Efficiency and Characterization of Vaccine Candidates

**Figure 7:**
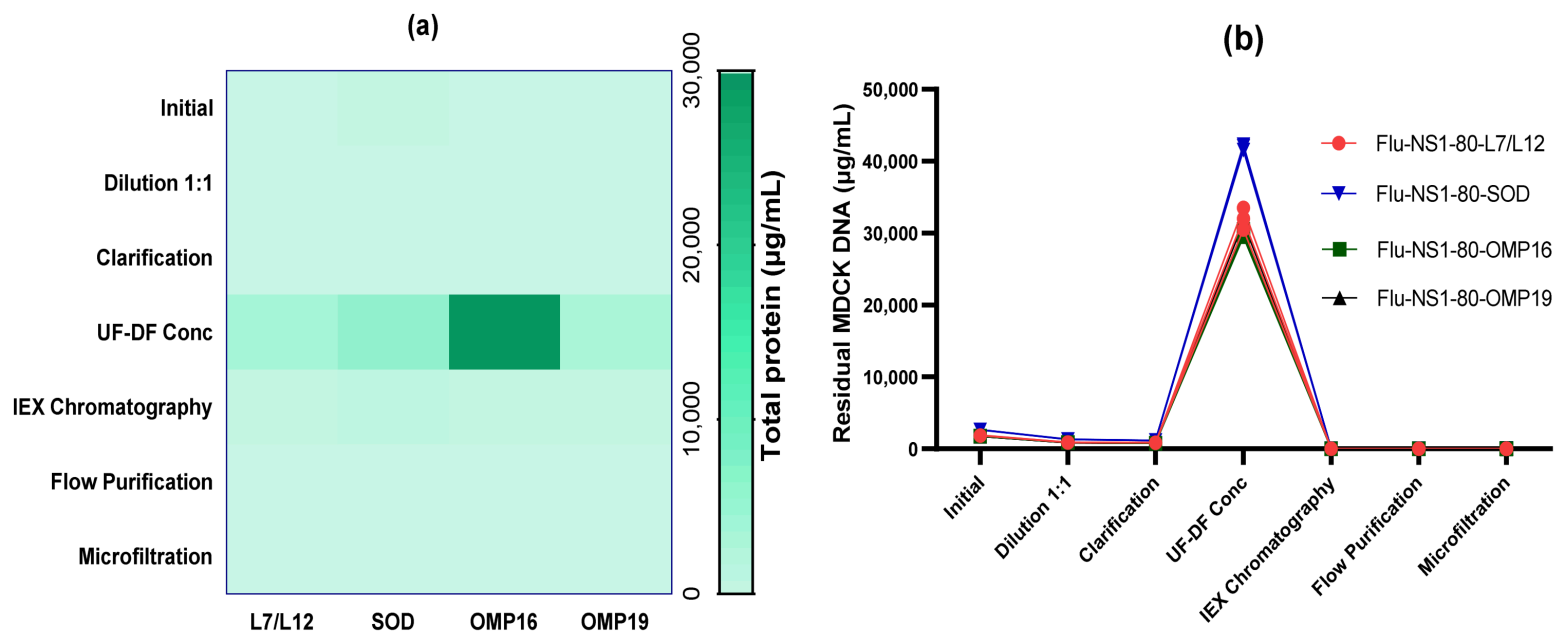
Comparative evaluation of impurity removal efficiency during the purification stages of recombinant influenza viruses. (**a**) Heatmap showing the dynamics of total protein concentration (μg/mL) across purification stages. Color intensity corresponds to protein concentration, as indicated by the vertical color scale bar on the right (ranging from 0 to 30,000 μg/mL), with light green/mint representing lower concentrations and dark green indicating higher concentrations. Purification stages (*Y*-axis): Initial material (Initial), Dilution 1:1, Clarification, UF/DF concentration (UF/DF Conc), Ion-exchange Chromatography (IEX Chromatography), Flow Purification, and Microfiltration. Strains (*X*-axis): L7/L12 (Flu-NS1-80-L7/L12), SOD (Flu-NS1-80-SOD), OMP16 (Flu-NS1-80-OMP16), OMP19 (Flu-NS1-80-OMP19). The darkest green signal at the UF/DF Conc stage, particularly for the OMP16 strain, reflects the temporary increase in total protein concentration due to a 20-fold volume reduction, while subsequent purification stages show progressive protein removal (lighter colors). (**b**) Residual host cell DNA. The *y*-axis represents the concentration of the corresponding parameter (logarithmic scale for DNA and endotoxins). Data are presented as mean ± SD (n = 3). The statistical significance of the parameter reduction was confirmed by ANOVA (*p* < 0.01).

The efficiency of removing impurities at each stage of the developed downstream workflow is illustrated in Figure 7.

As shown in the diagrams, the concentration step (UF/DF) was logically characterized by a temporary increase in the specific concentration of protein and DNA due to the 20-fold reduction in sample volume. However, subsequent anion-exchange chromatography on Q-Sepharose^®^ effectively separated the viral particles from the bulk of non-target host cell proteins: the protein concentration decreased by an average of 4.0–4.5-fold relative to the TFF concentrate. The most significant result of this step was the reduction of rHCD levels below the limit of detection (<10 ng/dose).

Final polishing on Capto™ Core 700 resin ensured the elimination of low-molecular-weight impurities and a further reduction in total protein concentration (by 4.5–4.8-fold relative to the ion-exchange chromatography step). Following the final sterile filtration, vaccine candidates were obtained with a protein concentration ranging from 125 ± 4 μg/mL (Flu-NS1-80-Omp16) to 175.1 ± 0.05 μg/mL (Flu-NS1-80-SOD) and bacterial endotoxin levels not exceeding 0.15 IU/mL.

All vaccine candidates met the stringent quality, purity, and safety criteria established for preparations intended for human use. In terms of infectious virus recovery, the final infectious titer ranged from 6.56 ± 0.39 to 7.47 ± 0.18 log_10_ TCID_50_/mL, with an overall cumulative recovery of infectious virus across the purification stages of 20–24%. Regarding purity, the final total protein content ranged from 125 ± 4 μg/mL to 175.1 ± 0.05 μg/mL, demonstrating a significant reduction in overall protein impurities, and residual host cell DNA concentrations were below the detection limit (<10 ng/dose) in all samples. In terms of safety, bacterial endotoxin levels ranged from 0.015 to 0.035 IU/mL—significantly below regulatory thresholds—and all samples were subjected to terminal sterile filtration to ensure microbiological purity. The genetic stability of the constructs and the presence of the target insert were confirmed by PCR and sequencing.

Thus, the developed downstream processing workflow ensures the production of viral vectors with high purification efficiency and predictable characteristics, supporting the use of this platform for further preclinical studies.

## 4. Discussion

This study demonstrates, for the first time, the feasibility of producing and purifying recombinant influenza A (H5N1) viral vectors expressing *Brucella* spp. antigens (Omp16, Omp19, L7/L12, and Cu–Zn SOD) at a pilot scale (20 L) using a fully scalable downstream processing (DSP) protocol that integrates membrane and chromatographic technologies. A key achievement of this work was the validation of the process across three independent pilot runs, confirming its high reproducibility and robustness for all four recombinant constructs (the inter-run coefficient of variation for yield did not exceed 10–12%). This indicates the high stability of the developed technological platform.

The obtained data confirm the rationale of a multi-stage purification strategy based on the sequential application of tangential flow filtration (TFF) and chromatography. Using TFF with 100 kDa molecular weight cut-off (MWCO) membranes effectively reduced the initial volume 20-fold (from 20 L to ~1.0–1.2 L), removed low-molecular-weight impurities (metabolites, salts, residual culture medium proteins), and standardized the buffer composition prior to chromatography. In modern bioprocessing, TFF is considered a preferred alternative to traditional concentration methods (e.g., polyethylene glycol precipitation or ultracentrifugation) due to its scalability, linear predictability, and gentle processing conditions [25]. These factors minimize the risk of viral inactivation, which is particularly crucial for influenza viruses, where preserving hemagglutinating and infectious activity is a key quality attribute (CQA).

Further separation and fine purification of the vectors were performed using a two-step chromatography approach: anion-exchange chromatography (AEX) on Q-Sepharose^®^ Fast Flow, followed by flow-through purification on the multimodal Capto™ Core 700 resin. During the primary capture step, the target virus bound firmly to the AEX matrix and was eluted as a narrow, symmetric peak using a stepwise salt gradient at 500 mM NaCl. This effectively separated viral particles from the bulk of host cell proteins and, crucially, reduced residual host cell DNA (rHCD) to levels below the limit of detection (<10 ng/dose). Final polishing was conducted in flow-through mode: due to the size-exclusion effect and multimodal ligands within the porous internal matrix of Capto™ Core 700, large influenza virions passed through the column unimpeded, while residual proteins and low-molecular-weight impurities were tightly retained inside the sorbent.

The use of advanced chromatographic materials for viral suspension purification aligns with cutting-edge trends in the DSP of enveloped viruses. As demonstrated by Banjac et al. (2014) [19], next-generation high-performance resins ensure deep removal of host cell impurities while fully preserving the structural and functional integrity of viral particles. Studies by Stein et al. (2020) and Oetomo et al. (2025) [26,27] emphasize that using flow-through sorbents during the polishing step minimizes virus-matrix contact time, thereby reducing the risk of conformational changes in surface glycoproteins. Thanks to optimized mass transfer mechanisms, such matrices feature high throughput, low hydrodynamic resistance, and the capacity to process large volumes without loss of resolution—qualities that make them highly attractive for scalable industrial purification compared to standard porous resins [28].

The developed two-step chromatographic approach demonstrated distinct technological advantages over classical sucrose density gradient ultracentrifugation. Despite its widespread use in laboratory practice [29], ultracentrifugation has significant limitations for industrial implementation: it is labor-intensive, poorly scalable, requires expensive specialized equipment, and poses a risk of partial virion destabilization under extreme centrifugal forces, often leading to particle aggregation and loss of biological activity. Furthermore, its limited throughput and batch-mode nature hinder its integration into modern GMP-compliant and continuous manufacturing lines. In contrast, our proposed combination of TFF and chromatography embodies the concept of gentle processing and offers direct prospects for process automation.

Of particular interest are the dynamics of hemagglutination activity (HA) during primary processing. The stability of this parameter after membrane clarification, followed by a titer increase to 7–8 log_2_ post-TFF, has a clear physicochemical rationale. Diafiltration across 100 kDa MWCO membranes effectively eliminates low-molecular-weight components of the culture medium (metabolites, ions, residual proteins) that can act as competitive inhibitors in the hemagglutination reaction. Extensive diafiltration against 5–10 diavolumes of phosphate buffer efficiently removes these bulk impurities. Consequently, the observed increase in HA post-TFF reflects not only viral concentration but also an enhancement in the functional integrity of the viral surface due to the removal of matrix inhibitors, thereby facilitating more efficient interaction between hemagglutinin and erythrocyte sialic acid receptors.

A comprehensive quantitative assessment of the process was conducted for four constructs: Flu-NS1-80-Omp16, Flu-NS1-80-Omp19, Flu-NS1-80-L7/L12, and Flu-NS1-80-Cu-Zn-SOD. During the initial stages (dilution and clarification), total protein concentration and rHCD content decreased proportionally to the dilution factor, optimizing the rheological properties of the material prior to ultrafiltration. The application of TFF achieved a 20-fold concentration alongside simultaneous buffer exchange.

The infectious titer of the final products ranged from 6.56 ± 0.39 to 7.47 ± 0.18 log_10_ TCID_50_/mL (fully satisfying the target criterion of ≥5.2 log_10_ TCID_50_/mL), and the cumulative recovery of infectious virus across the purification stages remained consistently at 20–24%. This infectious titer recovery aligns with current literature data for the multi-stage purification of sensitive enveloped viral vectors. Large-scale studies indicate that the recovery of infectious enveloped viruses in downstream processing typically ranges from 10% to 40% due to cumulative losses during matrix binding, elution, and ultrafiltration [29,30,31]. It should be noted that while laboratory-scale (flask) optimization was performed in our previous studies to establish baseline parameters, the current study specifically focuses on the validation and reproducibility of the process at the pilot scale (20 L), which is a critical translational step.

Importantly, the priority of our developed process was not to maximize infectious virus recovery at the expense of purity, but to ensure strict compliance with regulatory safety and quality criteria. In terms of product purity, the final total protein content ranged from 125 ± 4 μg/mL to 175.1 ± 0.05 μg/mL, demonstrating a significant reduction in overall protein impurities. While total protein quantification confirms the overall purification efficiency, specific quantification of host cell proteins (HCP) using validated immunochemical assays will be performed during the subsequent GMP validation phase. Regarding product safety, bacterial endotoxin levels ranged from 0.015 to 0.035 IU/mL—well below regulatory thresholds—and all preparations underwent terminal sterile filtration to minimize bioburden and ensure microbiological purity. The complete preservation of the target heterologous genetic insert in all final batches, confirmed by PCR and sequencing, demonstrates the high genetic stability of the engineered vectors under the intense hydrodynamic shear stress inevitably encountered during filtration and high-speed chromatography.

Despite these positive results, the study has certain limitations. First, the work was conducted at a pilot scale (20 L batch size); although all technologies used have proven scalability, final confirmation of process efficiency requires validation at an industrial scale. Second, this study focused on the technological parameters of in vitro downstream processing; the next critical step is to evaluate the immunogenicity and protective efficacy of these highly purified constructs in in vivo preclinical models. Furthermore, while the genetic integrity of the *Brucella* inserts was confirmed, specific immunological and structural characterization of the expressed heterologous antigens in the final purified product will be evaluated in the forthcoming in vivo preclinical models. Given the high yield, purity, and stability of the preparations, we plan to initiate preclinical trials in laboratory animals in the near future.

Collectively, the presented data demonstrate that the developed technological process ensures the production of recombinant viral vectors with high purification efficiency and predictable physicochemical and biological characteristics, meeting regulatory quality requirements for vaccine products. The platform has proven its flexibility (applicability to four different *Brucella* spp. antigens), robustness (validation across three independent runs), and scalability (compatibility with GMP requirements). These results establish a solid technological foundation for the translational development of candidate brucellosis vaccines into preclinical and clinical stages.

## 5. Conclusions

This study successfully developed and optimized a scalable, pilot-scale (20 L) downstream purification protocol for recombinant influenza A (H5N1) viral vectors expressing *Brucella* spp. antigens (Omp16, Omp19, L7/L12, and Cu–Zn SOD). The integrated platform, combining tangential flow filtration (TFF) with a two-step chromatographic approach (anion-exchange followed by multimodal flow-through polishing), demonstrated high reproducibility, robustness, and scalability. The final purified preparations consistently met stringent regulatory quality criteria, exhibiting high infectious titers (≥5.2 log_10_ TCID_50_/mL), minimal total protein content (125–175 μg/mL), residual host cell DNA below the detection limit (<10 ng/dose), and endotoxin levels well within safety thresholds (≤0.15 IU/mL). Importantly, the process preserved both the biological activity and the genetic integrity of the recombinant vectors. These results establish a solid technological foundation for the subsequent GMP-compliant manufacturing, preclinical evaluation, and clinical development of this promising candidate brucellosis vaccine.

## Data Availability

Data are contained within the article.
